# Population structure analysis and identification of genomic regions under selection associated with low-nitrogen tolerance in tropical maize lines

**DOI:** 10.1371/journal.pone.0239900

**Published:** 2020-09-29

**Authors:** Gustavo César Sant’Ana, Fernando Garcia Espolador, Ítalo Stefanine Correia Granato, Leandro Freitas Mendonça, Roberto Fritsche-Neto, Aluízio Borém

**Affiliations:** 1 Department of Agronomy, Universidade Federal de Viçosa, Viçosa, Minas Gerais, Brazil; 2 Department of Genetics, “Luiz de Queiroz” College of Agriculture, University of São Paulo, Piracicaba, São Paulo, Brazil; Murdoch University, AUSTRALIA

## Abstract

Increasing low nitrogen (N) tolerance in maize is an important goal for food security and agricultural sustainability. In order to analyze the population structure of tropical maize lines and identify genomic regions associated with low-N tolerance, a set of 64 inbred lines were evaluated under low-N and optimal-N conditions. The low-N Agronomic Efficiency index (LNAE) of each line was calculated. The maize lines were genotyped using 417,112 SNPs markers. The grouping based on the LNAE values classified the lines into two phenotypic groups, the first comprised by genotypes with high LNAE (named H_LNAE group), while the second one comprised genotypes with low LNAE (named L_LNAE group). The H_LNAE and L_LNAE groups had LNAE mean values of 3,304 and 1,644, respectively. The population structure analysis revealed a weak relationship between genetic and phenotypic diversity. Pairs of lines were identified, having at the same time high LNAE and high genetic distance from each other. A set of 29 SNPs markers exhibited a significant difference in allelic frequencies (Fst > 0.2) between H_LNAE and L_LNAE groups. The Pearson’s correlation between LNAE and the favorable alleles in this set of SNPs was 0.69. These SNPs could be useful for marker-assisted selection for low-N tolerance in maize breeding programs. The results of this study could help maize breeders identify accessions to be used in the development of low-N tolerant cultivars.

## Introduction

High levels of nitrogen (N) fertilization is required to achieve high maize (Zea mays L.) grain yield. However, the maize crop utilizes only about 30–40% of the N available in the soil while the remaining is lost through surface runoff, denitrification, volatilization and microbial consumption [1]. N fertilization is expensive and also harmful to the environment because of the negative effect on water quality. In this context, the development of nutritionally efficient maize cultivars, which yield more with low fertilization, is a way to reduce fertilizer usage [2, 3].

Maize tolerance to low availability of N in the soil is a complex trait influenced by genetic and environmental factors, including soil nitrogen availability, nitrogen uptake, assimilation, transportation, and remobilization [4]. Breeding programs use some indices related to low-N stress tolerance based on several quantitative traits [3, 5–8]. For example, Low-N Agronomic Efficiency (LNAE) is an index that accounts for the absolute grain yield at low-N and the ratio between low-N and optimal-N conditions [8]. Therefore, LNAE is an advantageous trait for maize breeding programs aimed at developing cultivars with high yield even at low-N availability.

Information on the genomic diversity and population structure of tropical maize germplasm can accelerate the genetic gains in maize breeding programs [9, 10]. Previous studies showed that maize gene pools present a clear population structure between temperate and tropical/subtropical pools. While the tropical maize germplasm has greater genetic diversity, the temperate germplasm presents more pronounced heterotic patterns [11–14]. However, the tropical maize germplasm lacks information on the genetic relationship [14] and genetic variation under low-N stress [8].

Maize lines from different heterotic groups produced higher-yielding hybrids than those of lines from the same heterotic group. Therefore, the assessment of genetic diversity of germplasm is routinely carried out using different morphological and molecular markers. For this purpose, molecular markers are more beneficial because they are not affected by environmental factors and the plants' developmental stage. Consequently, DNA markers have been an indispensable tool for characterizing genetic resources and providing breeders with more detailed information to assist in selecting diverse parents [9].

With the development of next-generation DNA sequencing methods, the Single Nucleotide Polymorphisms (SNPs) markers have become a useful tool for understanding genetic relationships and population structure of different species [15], including maize [9, 10, 14]. In addition, the genotyping of populations with high-density SNPs distributed throughout the maize genome enables the identification of genomic regions under directional selection among populations and associated with traits of interest [15, 16]. SNPs markers associated with traits of interest could benefit markers assisted selection pipelines of maize breeding programs.

The present study aimed i) at investigating the population structure and genomic diversity of selected tropical maize lines with different levels of tolerance to low-N stress, and ii) at identifying SNP markers under directional selection between groups of lines phenotypically contrasting in low-N tolerance.

## Material and methods

### Plant material and field experiments

A diversity panel consisting of 64 Brazilian tropical maize inbred lines with genetic variability for N-stress tolerance was evaluated. Most of these lines originated from the maize breeding program of the Universiade Federal de Viçosa (Brazil) [17, 18]. The maize lines were planted in two experimental fields, side by side, one with optimal-N availability (IN), and the other with low-N availability (LN). An 8 × 8 square lattice design with two replications was established in each field. The experiment was carried out at three sites (each combination of location and year was considered an environment): Anhembi, SP, Brazil (22°50’51” S, 48°01’06” W, 466 m) sown in the winter seasons of 2014 (site 1) and 2015 (site 2); and in Piracicaba, SP, Brazil (22°42’23” S, 47°38’14” W, 535 m) sown in the winter season of 2015 (site 3). The experimental units had 25 plants in a 5 m row with 0.80 m between rows.

The amount of N applied was determined by the expected yield using a given N dosage. Therefore, the IN dose corresponded to 100% expected yield and LN dose to 50% of IN yield [19]. In 2014 sites, the IN field was fertilized with 150 kg ha^−1^ of N while the LN field received 60 kg ha^−1^ of N. In 2015 sites, the doses were 100 kg ha^−1^ N and 35 kg ha^−1^ of N, respectively. The top-dressing doses were different from one year to another, but the expected yield ratio between the IN and LN was maintained [20].

### Traits evaluated

We measured the grain yield (GY, kg ha^-1^) of each plot and adjusted the yield to 13% grain moisture. The adjusted means were obtained for each inbred line under each nitrogen level and environment, using the following model:

y=Xβ+Zu+ϵ

where *y* is the grain yield vector, *β* was the vector of fixed effects of the common constant, the replicate, and the genotype, *u* was the vector of random effects of blocks with $u∼(0,I\sigma_{b}^{2})$, *ϵ* was the error matrix with *ϵ*∼(0,*Iσ*^2^), *X* and *Z* are the incidence matrix of the fixed and random effects, respectively.

Based on the adjusted means, we estimated the Low-N Agronomic Efficiency index (LNAE), for each inbred line in each environment. The LNAE index, introduced by [8], was calculated as follows:

$$LNAE_{ij}=\frac{GY_{LN_{ij}}}{GY_{IN_{ij}}}GY_{LN_{ij}}$$

where $GY_{IN_{ij}}$ and $GY_{LN_{ij}}$ are the same as defined earlier.

Analysis of variance was performed for LNAE index using the following model:

y=Xβ+ϵ

where *y* is the LNAE vector, *β* is the vector of fixed effects of the common constant, the environment, and the inbred line, *ϵ* is the error matrix with *ϵ*∼(0,*Iσ*^2^), *X* is the incidence matrix of the fixed effects. If there was significant effect of inbred line, we performed the Scott-Knott test using the package ‘Scott-Knott’ [21].

### Genotyping and SNPs quality control

Fresh leaves samples were collected from the fourth week old seedlings within each maize line and stored in a deep freezer at -80°C. According to its instruction manual, total genomic DNA extraction was performed using the Qiagen DNeasy Plant Mini Kit (Promega™). The inbred maize lines were genotyped using Affymetrix® Axiom® Maize Genotyping Array, containing 616,201 SNP markers [22]. The quality control of the SNPs was performed based on call rate (90%) and minor frequency allele (MAF, 5%) using TASSEL 5.0 [23] software. The quality control process resulted in 417,112 high-quality SNPs. From the total filtered SNPs, 12,050 were selected based on the LD pruning using Plink software [24], in order to remove neighbor markers possessing LD higher than 0.13, which was the average LD of the population [16].

### Population structure analysis

Population structure was analyzed using the Bayesian method implemented in STRUCTURE 2.3.4 [25] software, assuming an admixture model and independence between loci. After the initial burn-in period of 1 ×10^5^ iterations for each K value (ranging from 1 to 10) were performed ten replicate runs of 1 ×10^5^ Markov Chain Monte Carlo iterations. The STRUCTURE 2.3.4 results were summarized using the *pophelper* R package [26]. The number of groups was estimated using the Evanno’s ΔK based method [27]. Several runs for each K were submitted to the CLUMPP [28] to identify label switching.

In order to assess and visualize the genetic relationships among the maize lines, we performed principal coordinate analyses (PCoA) via genetic distance matrix with data standardization using the package *vegan* [29].

The genetic distance among the maize lines was calculated as 1 –IBS (identity by state) using TASSEL 5.0 [23]. IBS is defined as the probability that alleles sampled at random from two individuals at the same locus were the same.

### Detection of SNPs under selection between contrasting phenotypic groups

In order to identify SNPs with significant allele frequency differences between contrasting phenotypic groups for LNAE, we calculated the fixation index (Fst) for each SNP [30] using the SNiPlay [31] software. FsT values ranged from 0 to 1, with zero representing no allele differentiation and 1 representing complete allele differentiation between two populations. The SNPs possessing FsT > 0.2 were considered as under directional selection. We also used the Bayesian approach of the BAYESCAN software [32]. BAYESCAN was run with a burn-in of 50,000, a thinning interval of 30, a sample size of 5,000, a number of pilots runs of 50, length of pilot runs of 5,000, and the false discovery rate (FDR) threshold of 0.05.

## Results

### Genetic variability for low-N efficiency

**Fig 1:**
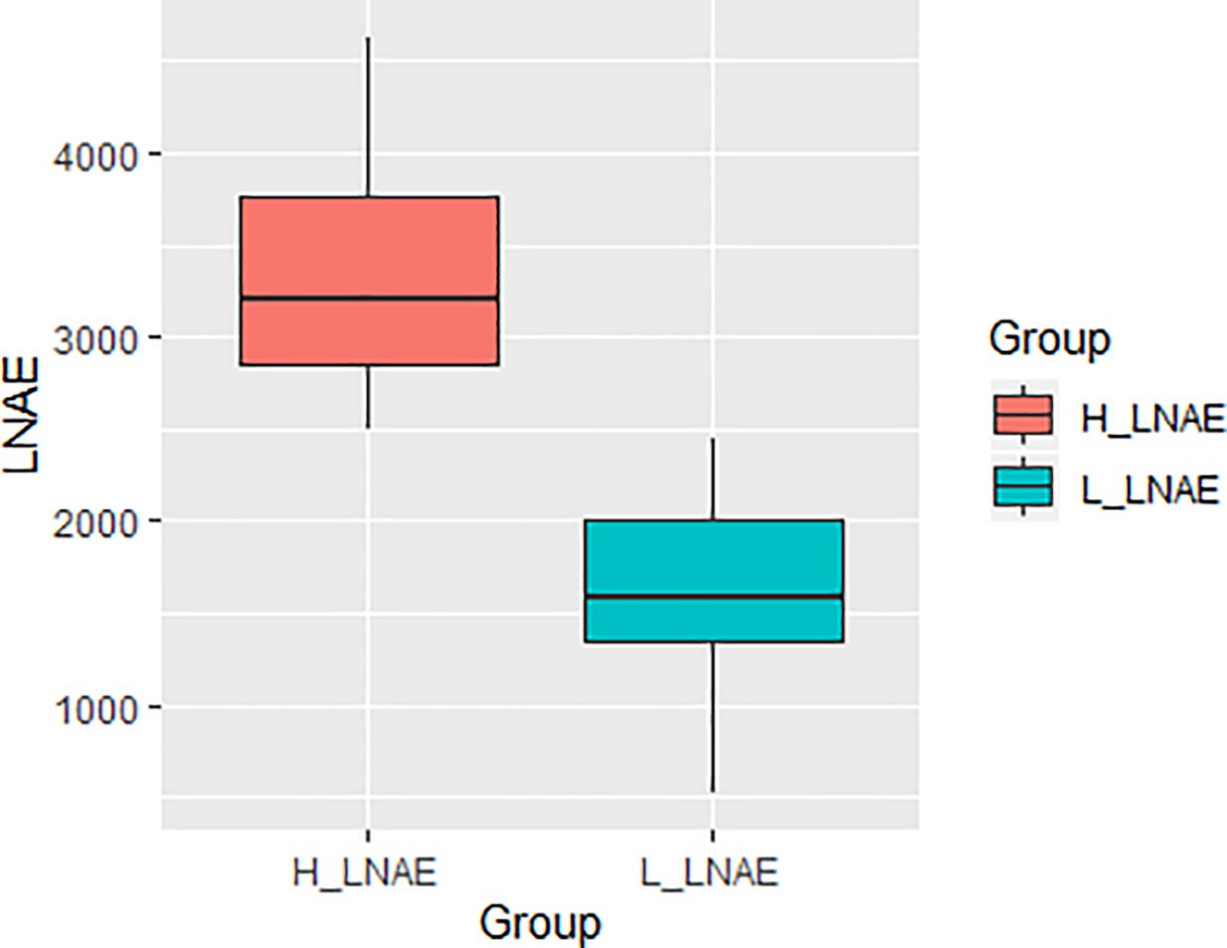
Box plots showing the distribution of the LNAE values of the high LNAE and low LNAE groups.

Based on the Scott-Knott test, the maize lines were classified into two phenotypic groups, one formed from 29 lines with high LNAE values (named H_LNAE group) and another comprising 35 lines with low LNAE (named L_LNAE group). The distribution of the LNAE values in the H_LNAE and L_LNAE groups is shown in Fig 1. The H_LNAE values of the genotypes ranged from 2,491 to 4,626, with an average and standard deviation of 3,304 and 589, respectively. The L_LNAE values of the lines ranged from 521 to 2448, with an average and standard deviation of 1,644 and 521, respectively. The LNAE values of the lines are shown in the S1 Table, while the phenotypic values for yield under low-N and optimal-N of each maize line in each site are available in S2 Table.

### Population structure and genetic distances using SNP markers

**Fig 2:**
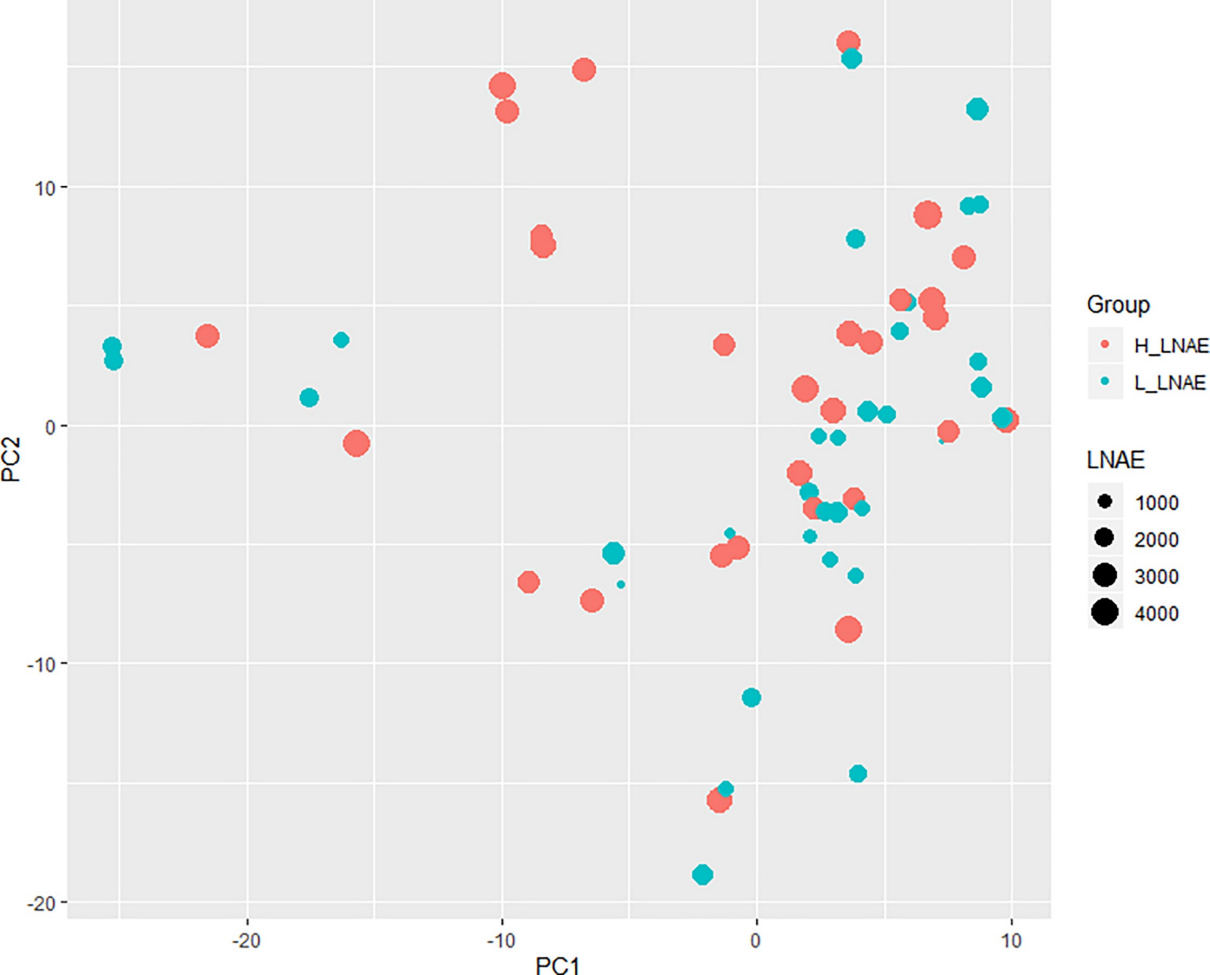
Principal coordinate analysis (PCoA) of 64 maize lines based on 12,050 polymorphic SNPs. The dots representing H_LNAE lines are colored red, while the Low L_LNAE lines are colored blue. The sizes of the dots are proportional to the LNAE values of the lines.

Principal coordinate analysis based on 12,050 SNPs markers (Fig 2) showed no clear genetic differentiation between H_LNAE and L_LNAE groups. The explanation for the lack of relationship between phenotypic and genetic differences is that the PCoA was obtained using all the 12,050 SNPs, several of which may have been may be positioned in genomic regions that do not affect the LNAE.

**Fig 3:**
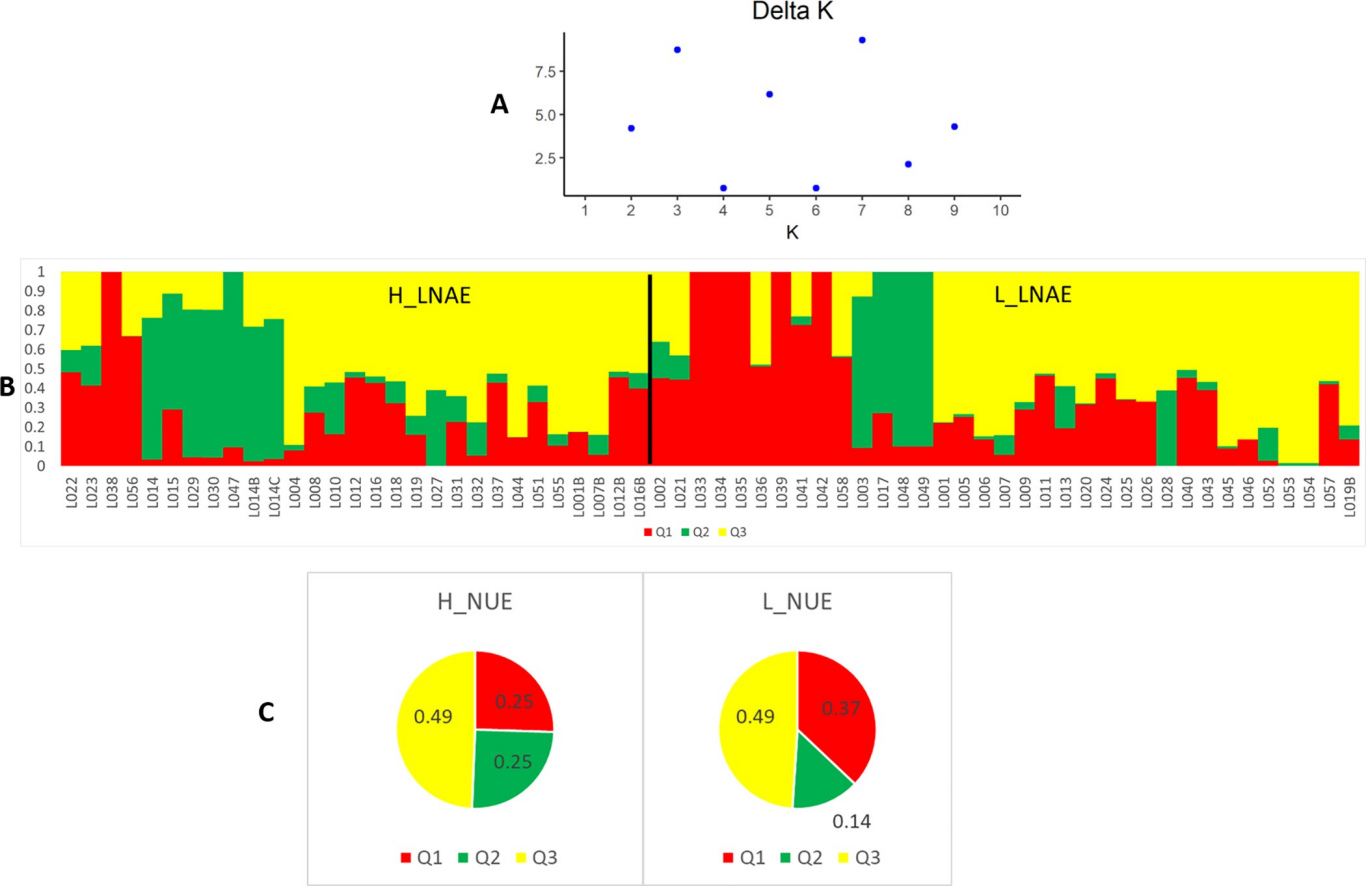
Bayesian population structure analysis of 64 tropical maize lines considering K = 3. (A) Delta K as a function of the number of groups. (B) Assignment of each line to Q1 (red), Q2 (green), and Q3 (yellow) genetic groups. (C) Joint assignment of H_LNAE and L_LNAE lines to Q1, Q2, and Q3 STRUCTURE groups.

According to the Bayesian approach, the population structure was analyzed using STRUCTURE software (Fig 3). Based on Evanno's criterion [27], the upper levels of the subdivision of the population were K = 7 and K = 3 (Fig 3A). Considering K = 3 (Fig 3B), the L_LNAE group presented a considerably higher assignment (Fig 3C) to Q1 (red) genetic group (0.37) than H_LNAE group (0.25). This indicated that although there was no clear genetic structure between the H_LNAE and L_LNAE groups, there were SNPs with significant differences in allelic frequencies between these groups.

The genetic distances among the 64 maize lines analyzed (S3 Table) ranged from 0.03 (between lines L033 and L039) to 0.41 (between lines L051 and L036). The line L51 showed a relatively high genetic distance in relation to all other lines analyzed (from 0.35 to 0.41). Considering only the group of lines possessing high LNAE (H_LNAE group), the genetic distances ranged from 0.05 (between L029 and L030) to 0.4 (between line L51 and lines L016, L012B, L037, L008, L012, L038, L022, L016B, L023, L056). The two lines with the highest LNAE values (L032 and L014) had a genetic distance of 0.24, indicating that crosses between them would be promising to generate transgressive hybrids. On the other hand, the cross between L029 and L030 lines is not very promising due to the high genetic similarity between them.

### Genomic regions under selection between H_LNAE and L_LNAE groups

**Fig 4:**
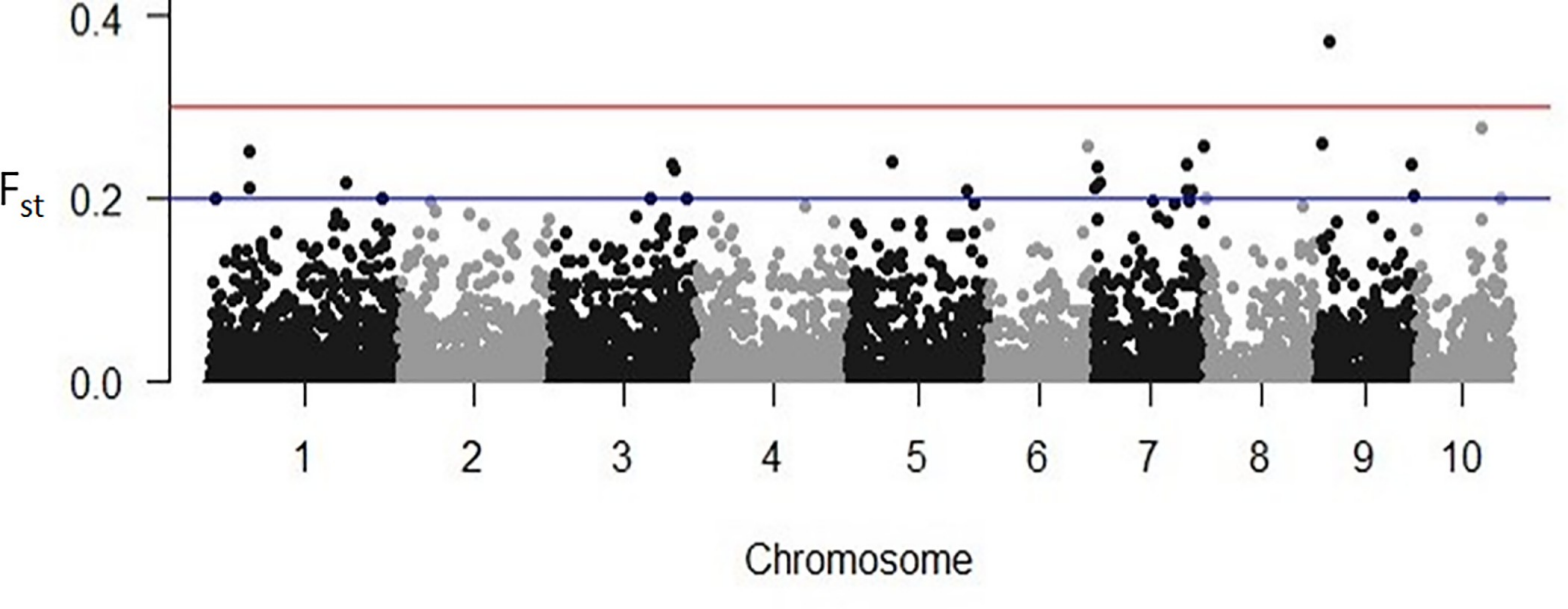
Fst values between H_LNAE and L_LNAE groups of the 12,050 SNPs distributed over the 10 maize chromosomes.

In order to identify SNPs under directional selection between H_LNAE and L_LNAE groups, we used two approaches, the first one based on Fst [30] and the second one using the BAYESCAN software [32]. 29 SNPs presented relatively high Fst (Fst> 0.2) in comparison to the overall average Fst (0.02). Fig 4 and Table 1 shows the markers, their positions, and their Fst values between H_LNAE and L_LNAE. The SNP Affx_90855476, located on chromosome 9, presented the highest Fst in relation to all other SNPs (0.37). Considering this SNP, the favorable allele for high LNAE (G) presented a frequency of 0.43 on the H_LNAE group, while in the group L_LNAE the frequency of this allele was only 0.03 (Table 2).

**Table 1:** The SNPs between H_LNAE and L_LNAE groups that show Fst values equal to or higher than 0.2.

SNP name	chromosome	Position (bp)	alleles	Fst
Affx_90711713	1	9443637	C/T	0.2
Affx_90909572	1	63413089	T/A	0.25
Affx_90186531	1	64972199	A/G	0.21
Affx_90356833	1	217334279	T/C	0.22
Affx_90534913	1	271904091	A/G	0.2
Affx_90553025	2	48427757	G/A	0.2
Affx_90385616	3	160016246	T/C	0.2
Affx_91290493	3	194998163	C/T	0.24
Affx_91132074	3	196436702	A/G	0.23
Affx_90368958	3	216007133	A/G	0.2
Affx_91170073	5	68421154	A/G	0.24
Affx_90587301	5	185404244	A/T	0.21
Affx_90387785	6	157988128	C/A	0.26
Affx_90507544	7	2957447	T/C	0.21
Affx_91297826	7	4239131	G/T	0.23
Affx_91199376	7	10182699	G/A	0.22
Affx_90364030	7	95121559	G/A	0.2
Affx_91068726	7	145152246	C/T	0.24
Affx_90988104	7	146589226	T/A	0.21
Affx_90071991	7	149946723	C/T	0.2
Affx_90384158	7	154184959	G/A	0.21
Affx_91148972	7	173957000	T/C	0.26
Affx_90225666	8	2103542	A/G	0.2
Affx_90795621	9	8699733	C/A	0.26
Affx_90855476	9	19161738	A/G	0.37
Affx_91232032	9	150818153	G/T	0.24
Affx_90678282	9	153416206	G/C	0.2
Affx_91206206	10	103426580	A/C	0.28
Affx_91335254	10	135327965	G/A	0.2

**Table 2:** SNPs under directional selection between H_LNAE and L_LNAE groups identified using BAYESCAN software. FA = favorable allele; FFA H_LNAE = frequency of the favorable alleles in the H_LNAE group; FFA L_LNAE = frequency of the favorable allele in the L_LNAE group.

SNP	chromosome	Position (bp)	alleles	FA	FFA H_LNAE	FFA L_LNAE
Affx_90387785	6	157988128	C/A	A	0.33	0.03
Affx_91199376	7	10182699	G/A	A	0.22	0
Affx_90855476	9	19161738	A/G	G	0.43	0.03
Affx_91232032	9	150818153	G/T	T	0.24	0

**Fig 5:**
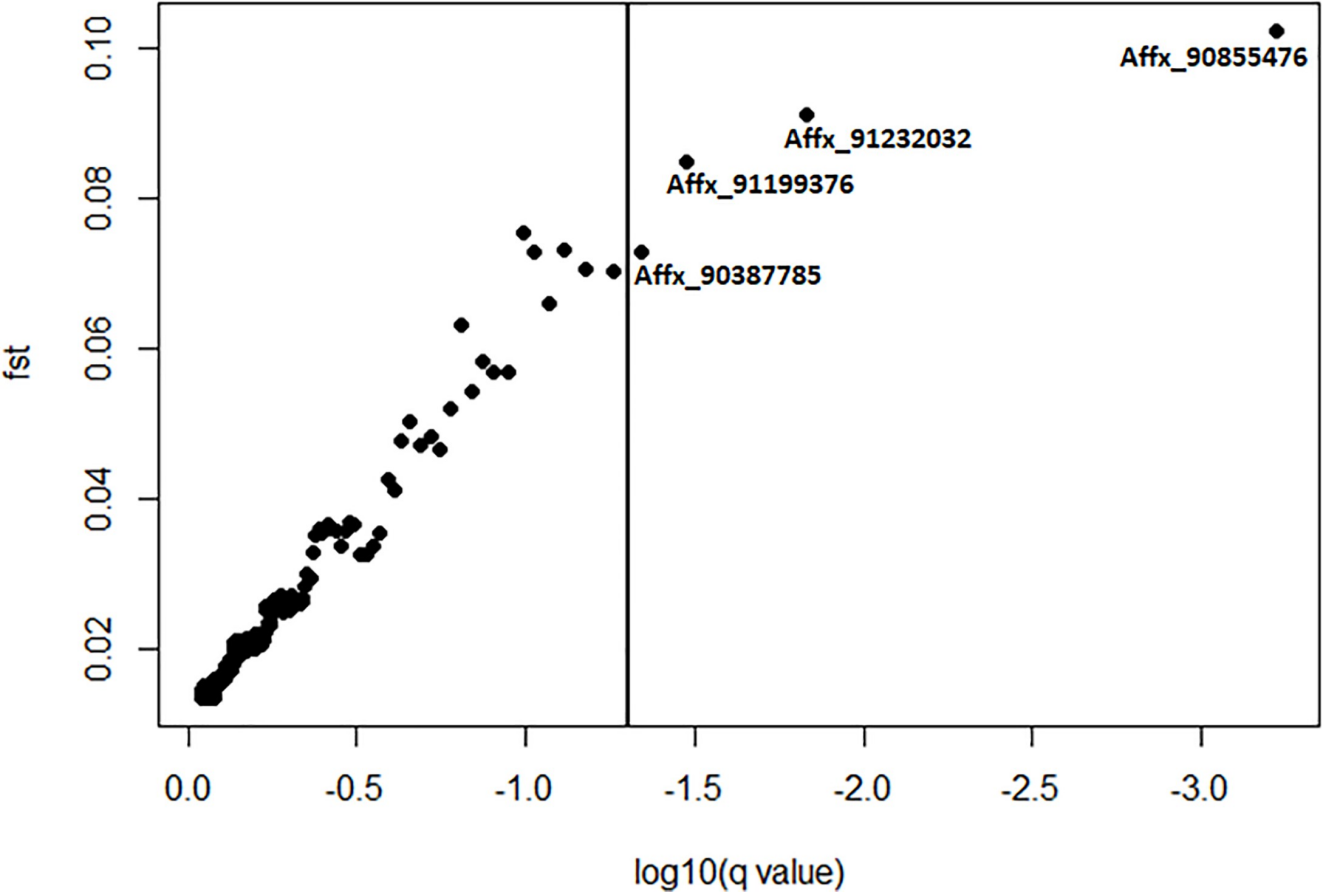
Plot representing the BAYESCAN results. The vertical line represents a false discovery rate (FDR) threshold of 0.05. Points to the right of the vertical line represent SNPs under directional selection between H_LNAE and L_LNAE groups.

We also used the method implemented in the BAYESCAN software to identify SNPs under directional selection between H_LNAE and L_LNAE groups. Through this approach, 4 SNPs were identified under selection between H_LNAE and L_LNAE groups (Fig 5 and Table 2).

**Fig 6:**
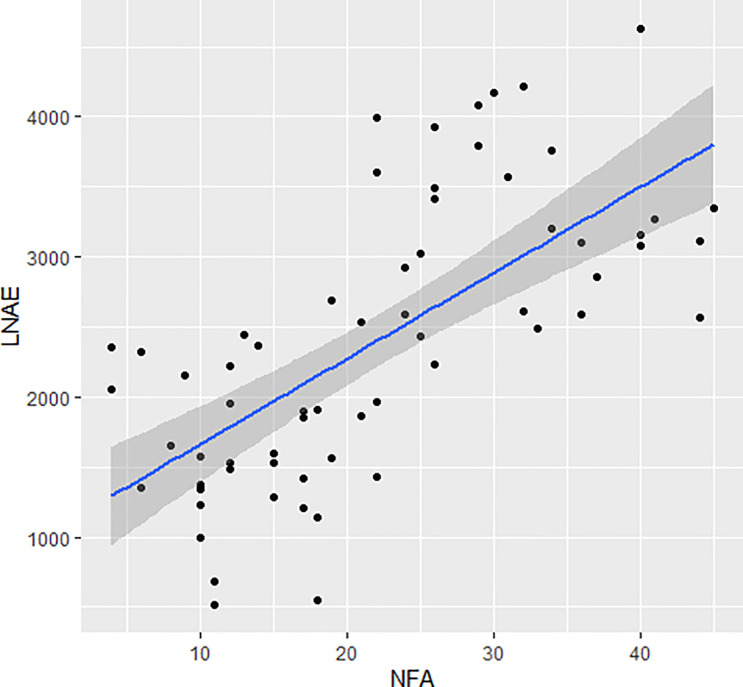
LNAE of 64 tropical maize lines as a function of the favorable alleles (NFA) in 29 SNPs with Fst > 0.2 between H_LNAE and L_LNAE groups.

Considering the 29 SNPs under selection between H_LNAE and L_LNAE groups, the Pearson's correlation between the number of favorable alleles (NFA) and the LNAE of the maize lines was 0.68 (p = 6.34 10^−10^). The R^2^ coefficient of LNAE linear regression as a function of NFA was 0.46 (Fig 6). The S4 Table shows the allelic profile of the maize lines for the 29 SNPs and the number of LNAE favorable alleles of each line.

## Discussion

Maize cultivars tolerant of low-N in the soil can show high grain yield with less N application. The possibility of applying smaller quantities of N in the maize crop is economically beneficial because it reduces production costs and is also environmentally favorable. In this study, we used the LNAE index (Low-N Agronomic Efficiency) to measure the tolerance of 64 tropical maize lines to low-N availability in the soil. In the LNAE index calculation, both the absolute GY at low-N availability and the ratio between low-N and optimum-N are considered [3, 8]. Therefore, LNAE is an important index in maize breeding programs aimed at developing high-yielding cultivars even under low-N conditions.

Genetic variability is essential for success in maize breeding programs. Therefore, genetic variability related to N-utilization has been investigated in maize, wheat, rice, and spring barley [2, 33–36]. In the present study, tropical maize lines analyzed possessed high diversity for low-N tolerance compared to studies previously reported [11, 37]. Thus, high LNAE inbred lines identified in this study may be an important resource for developing low-N tolerant cultivars.

A clear understanding of genetic relationships among low-N tolerant inbred lines offers an opportunity to obtain genotypes that could be used in parental combinations to develop simultaneously high-yielding and low-N tolerant maize hybrids. The genetic distance matrix (S3 Table) revealed a wide genetic variability among the maize lines analyzed, with a lower number of pairwise individuals possessing low genetic distances, suggesting that most of the tropical maize lines evaluated in this study were unique, and each of them aimed at developing the potential to contribute new alleles to the breeding programs in tropical regions.

That was possible to identify pairwise individuals that possessed high LNAE and were genetically distinct from each other. For example, the genetic distance between the two lines possessing the highest LNAE values (L032 and L014) was relatively high (0.24), indicating that the crossing between them would be promising and could result in transgressive hybrids. In addition, knowledge of genetic distances between pairs of maize lines can be useful for assigning lines to heterotic groups, selecting parental lines, and for estimating of genetic diversity loss during conservation or selection [9, 10, 12, 38, 39].

As a result of the development of next-generation DNA sequencing methods, SNPs molecular markers have become essential for understanding genetic relationships among different species of agronomic interest, including maize [10]. Additionally, genotyping populations with high-density SNPs distributed throughout the genome enabled the identification of SNPs associated with traits of interest, which broadened the knowledge about the genetic architecture of various traits of interest for maize breeding [16, 40, 41].

In practice, SNPs markers associated with traits can be beneficial when applied to assisted selection in breeding programs. However, from large-scale implementation in large breeding programs, the cost of genotyping large populations with dense SNP panels may still be a limiting factor in this tool [42–44]. Thus, selecting reduced sets of SNPs is important to reduce costs. Of the 417,112 polymorphic SNPs markers initially identified among the 64 tropical maize lines analyzed in the present study, we selected 12,050 SNPs based on linkage disequilibrium. These 12,050 SNPs were sufficient for estimation of the population structure of the lines efficiently. They allowed the identification of low-N tolerant lines and those with higher genetic distances to each other to maximize heterosis in future crosses among maize lines for hybrid production.

In order to generate an even smaller set of SNPs to use in marker-assisted selection for LNAE, we selected 29 SNPs with Fst values equal to or higher than 0.2 between the two phenotypic groups of maize lines possessing contrasting characteristics (H_LNAE and L_LNAE groups). We observed that four of these SNPs (Affx_90387785, Affx_91199376, Affx_90855476, and Affx_91232032) also showed evidence of having undergone directional selection according to the results of the analysis carried out using the BAYESCAN software [32]. BAYESCAN is based on the multinomial-Dirichlet model. As Bayesian, BAYESCAN incorporates the uncertainty of allele frequencies due to small sample sizes. The false discovery rate threshold adopted in this analysis was considerably low (0.05). Therefore, the number of SNPs under directional selection identified using this approach was lower than using the Fst [30] based approach.

Considering the small set of 29 SNPs under selection between H_LNA and L_LNAE groups, we observed a Pearson’s correlation between the number of favorable alleles for high LNAE and the LNAE index of the maize lines was equal to 0.68. These SNP markers can be useful in marker-assisted selection for low-N tolerance. In addition, the results can help establish breeding programs to improve tolerance of maize to stress due to the low availability of N in the soil.

## Supporting information

S1 Table Low-N Agronomic Efficiency (LNAE) index (best linear unbiased estimates) of the 64 tropical maize lines analyzed in this study. (XLSX)

S2 Table LNAE and grain yield of 64 tropical maize lines under low-N and optimal-N conditions in three environments. (XLSX)

S3 Table Genetic distance matrix among 64 tropical maize lines. (XLSX)

S4 Table Allele profiles of the 64 tropical maize lines for the 29 SNPs possessing Fst > 0.2 between the phenotypically contrasting groups (L_LNAE and H_LNAE groups) and number of favorable alleles for LNAE that each line possess. (XLSX)

## References

[1] Kant S, Bi YM, Rothstein SJ. Understanding plant response to nitrogen limitation for the improvement of crop nitrogen use efficiency. Vol. 62, Journal of Experimental Botany. 2011 p. 1499–509. 10.1093/jxb/erq297 20926552

[2] Cui Z, Chen X, Zhang F. Current Nitrogen management status and measures to improve the intensive wheat-maize system in China. Ambio. 2010;39(6):376–84. 21053721 10.1007/s13280-010-0076-6 PMC3357710

[3] Mendonça L de F, Granato ÍSC, Alves FC, Morais PPP, Vidotti MS, Fritsche-Neto R. Accuracy and simultaneous selection gains for N-stress tolerance and N-use efficiency in maize tropical lines. Sci Agric [Internet]. 2017 12;74(6):481–8. Available from: http://www.scielo.br/scielo.php?script=sci_arttext&pid=S0103-90162017000600481&lng=en&tlng=en

[4] Xu G, Fan X, Miller AJ. Plant Nitrogen Assimilation and Use Efficiency. Annu Rev Plant Biol [Internet]. 2012 6 2;63(1):153–82. Available from: http://www.annualreviews.org/doi/10.1146/annurev-arplant-042811-105532 22224450 10.1146/annurev-arplant-042811-105532

[5] Craswell ET, Godwin DC. The efficiency of nitrogen fertilizers applied to cereals grown in different climates In: Tinker PB, Lauchli A, editors. Advances in plant nutrition. New York: Praeger Publishers; 1984 p. 1–55.

[6] Resende MDV de, Duarte JB. Precisão E Controle De Qualidade Em Experimentos De Avaliação De Cultivares. Pesqui Agropecuária Trop (Agricultural Res Trop). 2007;37(3):182–94.

[7] Miti F, Pangirayi T, Derera J. S1 selection of local maize landraces for low soil nitrogen tolerance in Zambia. African J Plant Sci [Internet]. 2010;4(3):67–81. Available from: http://www.academicjournals.org/article/article1380108743_Miti et al.pdf

[8] Wu Y, Liu W, Li X, Li M, Zhang D, Hao Z, et al Low-nitrogen stress tolerance and nitrogen agronomic efficiency among maize inbreds: comparison of multiple indices and evaluation of genetic variation. Euphytica [Internet]. 2011 7 16;180(2):281–90. Available from: http://link.springer.com/10.1007/s10681-011-0409-y

[9] Boakyewaa Adu G, Badu-Apraku B, Akromah R, Garcia-Oliveira AL, Awuku FJ, Gedil M. Genetic diversity and population structure of early-maturing tropical maize inbred lines using SNP markers. Kalendar R, editor. PLoS One [Internet]. 2019 4 9;14(4):e0214810 Available from: 10.1371/journal.pone.0214810 30964890 PMC6456193

[10] Romay MC, Millard MJ, Glaubitz JC, Peiffer JA, Swarts KL, Casstevens TM, et al Comprehensive genotyping of the USA national maize inbred seed bank. Genome Biol [Internet]. 2013 6 11;14(6):R55 Available from: http://genomebiology.com/content/pdf/gb-2013-14-6-r55.pdf 23759205 10.1186/gb-2013-14-6-r55 PMC3707059

[11] Lu Y, Yan J, Guimarães CT, Taba S, Hao Z, Gao S, et al Molecular characterization of global maize breeding germplasm based on genome-wide single nucleotide polymorphisms. Theor Appl Genet [Internet]. 2009 12 11;120(1):93–115. Available from: http://link.springer.com/10.1007/s00122-009-1162-7 19823800 10.1007/s00122-009-1162-7

[12] Semagn K, Magorokosho C, Vivek BS, Makumbi D, Beyene Y, Mugo S, et al Molecular characterization of diverse CIMMYT maize inbred lines from eastern and southern Africa using single nucleotide polymorphic markers. BMC Genomics [Internet]. 2012;13(1):1–11. Available from: http://bmcgenomics.biomedcentral.com/articles/10.1186/1471-2164-13-113 22443094 10.1186/1471-2164-13-113 PMC3362765

[13] Mir C, Zerjal T, Combes V, Dumas F, Madur D, Bedoya C, et al Out of America: tracing the genetic footprints of the global diffusion of maize. Theor Appl Genet [Internet]. 2013 11 7;126(11):2671–82. Available from: http://link.springer.com/10.1007/s00122-013-2164-z 23921956 10.1007/s00122-013-2164-z

[14] Andrade LRB de, Fritsche Neto R, Granato ÍSC, Sant’Ana GC, Morais PPP, Borém A. Genetic Vulnerability and the Relationship of Commercial Germplasms of Maize in Brazil with the Nested Association Mapping Parents. Chen C, editor. One PLoS [Internet]. 2016 10 25;11(10):1–14. Available from: http://dx.plos.org/10.1371/journal.pone.0163739 10.1371/journal.pone.0163739 PMC5079593 27780247

[15] Sant’Ana GC, Pereira LFP, Pot D, Ivamoto ST, Domingues DS, Ferreira R V., et al Genome-wide association study reveals candidate genes influencing lipids and diterpenes contents in Coffea arabica L. Sci Rep [Internet]. 2018 12 11;8:465 Available from: http://www.nature.com/articles/s41598-017-18800-1 29323254 10.1038/s41598-017-18800-1 PMC5764960

[16] Morosini JS, Mendonça L de F, Lyra DH, Galli G, Vidotti MS, Fritsche-Neto R. Association mapping for traits related to nitrogen use efficiency in tropical maize lines under field conditions. Plant Soil [Internet]. 2017 12 2;421(1–2):453–63. Available from: http://link.springer.com/10.1007/s11104-017-3479-3

[17] Lanes ECM, Viana JMS, Paes GP, Paula MFB, Maia C, Caixeta ET, et al Population structure and genetic diversity of maize inbreds derived from tropical hybrids. Genet Mol Res [Internet]. 2014;13(3):7365–76. Available from: http://www.funpecrp.com.br/gmr/year2014/vol13-3/pdf/gmr3251.pdf 10.4238/2014.September.12.2 25222235

[18] Lanes ÉCM de. Caracterização molecular de linhagens de milho tropical por marcadores microssatélites. Universidade Federal de Viçosa; 2010.

[19] Gallais A, Hirel B. An approach to the genetics of nitrogen use efficiency in maize. J Exp Bot [Internet]. 2004 2 1;55(396):295–306. Available from: https://academic.oup.com/jxb/article-lookup/doi/10.1093/jxb/erh006 14739258 10.1093/jxb/erh006

[20] Raij B van, Cantarela H, Quaggio JA, Furlani ÂMC, editors. Boletim Técnico 100—Recomendações de adubação e calagem para o estado de São Paulo. 2nd ed Campinas; 1997. 285 p.

[21] Jelihovschi E, Faria JC, Allaman IB. ScottKnott: A Package for Performing the Scott-Knott Clustering Algorithm in R. TEMA (São Carlos) [Internet]. 2014 3 5;15(1):3–17. Available from: http://tema.sbmac.org.br/tema/article/view/646

[22] Unterseer S, Bauer E, Haberer G, Seidel M, Knaak C, Ouzunova M, et al A powerful tool for genome analysis in maize: development and evaluation of the high density 600 k SNP genotyping array. BMC Genomics [Internet]. 2014;15(1):823 Available from: http://bmcgenomics.biomedcentral.com/articles/10.1186/1471-2164-15-823 25266061 10.1186/1471-2164-15-823 PMC4192734

[23] Bradbury PJ, Zhang Z, Kroon DE, Casstevens TM, Ramdoss Y, Buckler ES. TASSEL: Software for association mapping of complex traits in diverse samples. Bioinformatics. (2007); 23:2633–2635. Available from: 10.1093/bioinformatics/btm308 17586829

[24] Purcell S, Neale B, Todd-Brown K, Thomas L, Ferreira MAR, Bender D, et al PLINK: A Tool Set for Whole-Genome Association and Population-Based Linkage Analyses. Am J Hum Genet [Internet]. 2007 9;81(3):559–75. Available from: https://linkinghub.elsevier.com/retrieve/pii/S0002929707613524 10.1086/519795 17701901 PMC1950838

[25] Pritchard JK, Stephens M, Donnelly P. Inference of Population Structure Using Multilocus Genotype Data. Genetics. 2000;155:945–59. 10835412 10.1093/genetics/155.2.945 PMC1461096

[26] Francis RM. pophelper: an R package and web app to analyse and visualize population structure. Mol Ecol Resour [Internet]. 2017 1;17(1):27–32. Available from: http://doi.wiley.com/10.1111/1755-0998.12509 26850166 10.1111/1755-0998.12509

[27] Evanno G, Regnaut S, Goudet J. Detecting the number of clusters of individuals using the software STRUCTURE: A simulation study. Mol Ecol. 2005;14(8):2611–20. 10.1111/j.1365-294X.2005.02553.x 15969739

[28] Jakobsson M, Rosenberg NA. CLUMPP: a cluster matching and permutation program for dealing with label switching and multimodality in analysis of population structure. Bioinformatics [Internet]. 2007;23(14):1801–6. Available from: https://academic.oup.com/bioinformatics/article-lookup/doi/10.1093/bioinformatics/btm233 17485429 10.1093/bioinformatics/btm233

[29] Oksanen J, Blanchet FG, Kindt R, Legendre P, Minchin PR, O’Hara RB, et al. Package vegan: Community Ecology Package [Internet]. R package version 2.3–1. 2013. Available from: http://cran.r-project.org/package=vegan

[30] Weir BS, Cockerham CC. Estimating F-Statistics for the Analysis of Population Structure. Evolution (N Y) [Internet]. 1984 11;38(6):1358 Available from: https://www.jstor.org/stable/2408641?origin=crossref 10.1111/j.1558-5646.1984.tb05657.x 28563791

[31] Dereeper A, Homa F, Andres G, Sempere G, Sarah G, Hueber Y, et al SNiPlay3: a web-based application for exploration and large scale analyses of genomic variations. Nucleic Acids Res [Internet]. 2015 7 1;43(W1):W295–300. Available from: https://academic.oup.com/nar/article-lookup/doi/10.1093/nar/gkv351 26040700 10.1093/nar/gkv351 PMC4489301

[32] Foll M, Gaggiotti O. A Genome-Scan Method to Identify Selected Loci Appropriate for Both Dominant and Codominant Markers: A Bayesian Perspective. Genetics [Internet]. 2008 10;180(2):977–93. Available from: http://www.genetics.org/lookup/doi/10.1534/genetics.108.092221 18780740 10.1534/genetics.108.092221 PMC2567396

[33] Moll RH, Kamprath EJ, Jackson WA. Analysis and Interpretation of Factors Which Contribute to Efficiency of Nitrogen Utilization. Agron J. 1982;74(3):562.

[34] Cox MC, Qualset CO, Rains DW. Genetic Variation for Nitrogen Assimilation and Translocation in Wheat. II. Nitrogen Assimilation in Relation to Grain Yield and Protein. Crop Sci [Internet]. 1985;25(3):430–40. Available from: https://www.crops.org/publications/cs/abstracts/25/3/CS0250030435

[35] Singh U, Ladha JK, Castillo EG, Punzalan G, Tirol-padre A, Duqueza M. Genotypic variation in nitrogen use efficiency in medium- and long-duration rice. F Crop Res. 1998;58:35–53.

[36] Anbessa Y, Juskiw P, Good A, Nyachiro J, Helm J. Genetic Variability in Nitrogen Use Efficiency of Spring Barley. Crop Sci [Internet]. 2009;49(4):1259 Available from: https://www.crops.org/publications/cs/abstracts/49/4/1259

[37] Li T, Qu J, Wang Y, Chang L, He K, Guo D, et al Genetic characterization of inbred lines from Shaan A and B groups for identifying loci associated with maize grain yield. BMC Genet [Internet]. 2018 12 23;19(63):1–12. Available from: https://bmcgenet.biomedcentral.com/articles/10.1186/s12863-018-0669-9 30139352 10.1186/s12863-018-0669-9 PMC6108135

[38] Reif JC, Melchinger AE, Xia XC, Warburton ML, Hoisington DA, Vasal SK, et al Genetic Distance Based on Simple Sequence Repeats and Heterosis in Tropical Maize Populations. Crop Sci [Internet]. 2003;43(4):1275–82. Available from: https://www.crops.org/publications/cs/abstracts/43/4/1275

[39] Tian F, Bradbury PJ, Brown PJ, Hung H, Sun Q, Flint-Garcia S, et al Genome-wide association study of leaf architecture in the maize nested association mapping population. Nat Genet. 2011;43(2):159–62. 10.1038/ng.746 21217756

[40] Zhu XM, Shao XY, Pei YH, Guo XM, Li J, Song XY, et al Genetic diversity and genome-wide association study of major ear quantitative traits using high-density SNPs in maize. Front Plant Sci. 2018;9:1–16. 10.3389/fpls.2018.00001 30038634 PMC6046616

[41] Mazaheri M, Heckwolf M, Vaillancourt B, Gage JL, Burdo B, Heckwolf S, et al Genome-wide association analysis of stalk biomass and anatomical traits in maize. BMC Plant Biol. 2019;19:1–17. 10.1186/s12870-018-1600-2 30704393 PMC6357476

[42] VanRaden PM, O’Connell JR, Wiggans GR, Weigel KA. Genomic evaluations with many more genotypes. Genet Sel Evol [Internet]. 2011;43(10):1–11. Available from: http://www.gsejournal.org/content/43/1/10 10.1186/1297-9686-43-10 PMC3056758 21366914

[43] Carvalheiro R, Boison SA, Neves HHR, Sargolzaei M, Schenkel FS, Utsunomiya YT, et al Accuracy of genotype imputation in Nelore cattle. Genet Sel Evol. 2014;46(1):1–11. 10.1186/s12711-014-0069-1 PMC4192291 25927950

[44] Yoshida GM, Lhorente JP, Correa K, Soto J, Salas D, Yáñez JM. Genome-wide association study and cost-efficient genomic predictions for growth and fillet yield in Nile tilapia (Oreochromis niloticus). G3 Genes, Genomes, Genet. 2019;9(8):2597–607. 10.1534/g3.119.400116 PMC6686944 31171566

